# Probing Activation and Conformational Dynamics of the Vesicle-Reconstituted β_2_ Adrenergic Receptor at the Single-Molecule Level

**DOI:** 10.1021/acs.jpcb.3c08349

**Published:** 2024-02-23

**Authors:** Marijonas Tutkus, Christian V. Lundgaard, Salome Veshaguri, Asger Tønnesen, Nikos Hatzakis, Søren G. F. Rasmussen, Dimitrios Stamou

**Affiliations:** † Department of Chemistry, University of Copenhagen, Universitetsparken 5, DK-2100 Copenhagen, Denmark; ‡ Department of Neuroscience and Pharmacology, Panum, University of Copenhagen, Blegdamsvej 3, DK-2200 Copenhagen, Denmark; § Center for Geometrically Engineered Cellular Systems, Universitetsparken 5, DK-2100 Copenhagen, Denmark; ∥ Department of Chemistry and Nanoscience Center, University of Copenhagen, Universitetsparken 5, DK-2100 Copenhagen, Denmark; ⊥ Institute of Biotechnology, Life Sciences Center, Vilnius University, Saulėtekio Ave. 7, LT-10257 Vilnius, Lithuania; # Department of Molecular Compound Physics, Center for Physical Sciences and Technology, Saulėtekio Ave. 3, LT-10257 Vilnius, Lithuania

## Abstract

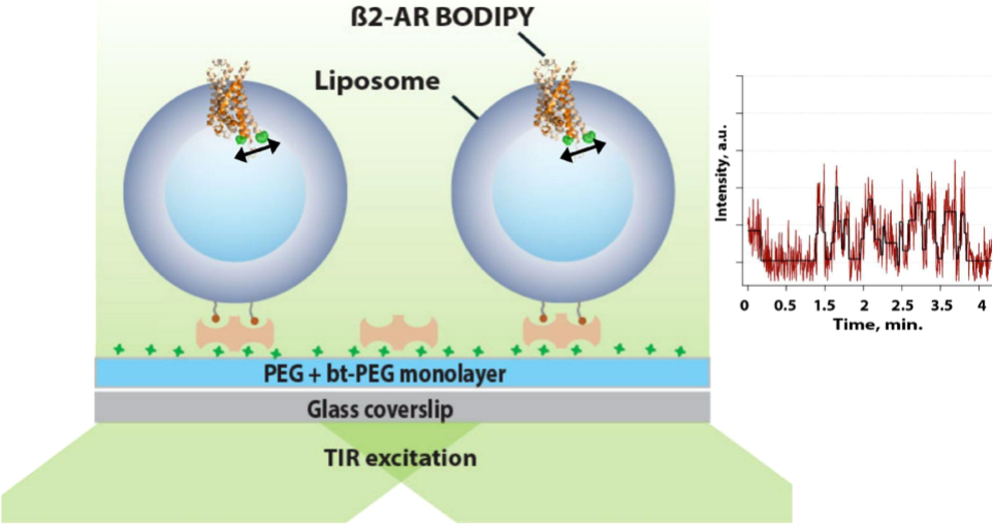

G-protein-coupled receptors (GPCRs) are structurally flexible membrane proteins that mediate a host of physiological responses to extracellular ligands like hormones and neurotransmitters. Fine features of their dynamic structural behavior are hypothesized to encode the functional plasticity seen in GPCR activity, where ligands with different efficacies can direct the same receptor toward different signaling phenotypes. Although the number of GPCR crystal structures is increasing, the receptors are characterized by complex and poorly understood conformational landscapes. Therefore, we employed a fluorescence microscopy assay to monitor conformational dynamics of single β_2_ adrenergic receptors (β_2_ARs). To increase the biological relevance of our findings, we decided not to reconstitute the receptor in detergent micelles but rather lipid membranes as proteoliposomes. The conformational dynamics were monitored by changes in the intensity of an environmentally sensitive boron-dipyrromethene (BODIPY 493/503) fluorophore conjugated to an endogenous cysteine (located at the cytoplasmic end of the sixth transmembrane helix of the receptor). Using total internal reflection fluorescence microscopy (TIRFM) and a single small unilamellar liposome assay that we previously developed, we followed the real-time dynamic properties of hundreds of single β_2_ARs reconstituted in a native-like environment—lipid membranes. Our results showed that β_2_AR-BODIPY fluctuates between several states of different intensity on a time scale of seconds, compared to BODIPY-lipid conjugates that show almost entirely stable fluorescence emission in the absence and presence of the full agonist BI-167107. Agonist stimulation changes the β_2_AR dynamics, increasing the population of states with higher intensities and prolonging their durations, consistent with bulk experiments. The transition density plot demonstrates that β_2_AR-BODIPY, in the absence of the full agonist, interconverts between states of low and moderate intensity, while the full agonist renders transitions between moderate and high-intensity states more probable. This redistribution is consistent with a mechanism of conformational selection and is a promising first step toward characterizing the conformational dynamics of GPCRs embedded in a lipid bilayer.

## Introduction

Most GPCRs bind signaling molecules on the extracellular surface and interact with other proteins on the intracellular surface in this way to form a signaling hub.^1^ Ligand binding initiates conformational changes that propagate from the extracellular surface to the intracellular surface of the receptor. Therefore, signal transduction mediated by GPCRs is strictly coupled to a conformational change. Activation of the receptor stimulates G-proteins to bind the intracellular surface of GPCRs.^2,3^ The bound G-protein becomes activated, triggering signaling cascades.^4^

β_2_AR is a class A GPCR; it preferentially couples to Gs over Gi but does not couple Gq.^5^ This and other related receptors are highly dynamic structures, and even under basal conditions (i.e., in the absence of any ligands), they exist in multiple conformations.^6^ Ligand binding shifts the conformational equilibrium of the receptor, modulating their function. Such observations were made possible by synthetic ligands with efficacy profiles ranging from inverse agonists (suppress basal activity) to full agonists (promote activation of Gs).^7^ It is intriguing how structurally similar ligands can drive the conformational ensemble to various different substates and cause diverse responses.^8^

Recent crystallographic structures of distinct class A GPCRs at different conformations reveal the mechanism of activation.^9−12^ They show that the largest conformational change associated with receptor activation is an outward movement of the cytoplasmic end of the sixth transmembrane helix (TM6). Ensemble techniques reveal that even the most potent agonists fail to fully stabilize β_2_AR in its activated conformation without G-protein or stabilizing nanobodies present.^13−16^ Thus, we require inherently dynamic techniques that monitor transient species to get a better picture of active-state conformational dynamics. An older study demonstrated that a single-fluorescein dye molecule coupled to the TM6 (at C265) of a full-length β_2_AR mutant (G224L, C378A, and C406A) monitors conformational substates of this receptor in detergent micelles.^17^ Recent ground-breaking single-molecule (SM) study of the full-length minimal cysteine ß_2_AR-TMR (TMR at C265) mutant in a detergent environment shows that the fluorescence of a single-dye labeled ß_2_AR shifts its intensity-lifetime upon full agonist binding.^18^ A ground-breaking study of the full-length minimum cysteine β_2_AR mutant with a Förster resonance energy transfer (FRET) pair label in detergent micelles reports the movements of TM6 in the presence of ligands.^19^ It shows that the ligands have distinct efficacy profiles and have distinct effects on receptor structure, dynamics, and G-protein coupling.^19^ TM6 of a Cy3-labeled β_2_AR (Cy3 at Cys265; mutations E122W, C265A and C341A, truncation beyond residue 348, and removal of residues 245–249) in lipid nanodiscs also fluctuate with distinct efficacy profiles.^20^ In a further study of β_2_AR (mutations E122W, C327S and C341A, truncation beyond residue 348, and removal of residues 245–249) in nanodiscs, movements of TM7 were probed by Cy3 dye placed at residue Cys327.^21^ There is an apparent discrepancy between time scales of conformational dynamics reported previously^18−20^ and physiological responses measured in living cells.^22^ The reason behind this is unclear: One possibility is that everything happens differently in intact cells. It may be related to precoupling of G-proteins to receptors, which does not occur in our experiments. For the small number of receptors that are precoupled to G-proteins in the cell, it is unlikely that the duration of the state of unliganded receptors limits how fast they can be interconverted into an active conformation upon ligand binding. Another possibility is that the duration of states does not determine the interconversion rate.

Here, we focused on characterizing the SM dynamics of the β_2_AR when reconstituted in a lipid environment. The β_2_AR receptor in our study was truncated at C365, labeled at the end of TM6 on residue C265 with BODIPY 493/503, and was reconstituted in proteoliposomes. Proteoliposomes were immobilized on a biotin-polyethylene glycol-poly-l-Lysine-functionalized (biotin–PEG-PLL) surface via neutravidin and imaged with TIRFM.^23−25^ In previous work, purified β_2_AR was labeled at Cys265 with a variety of cysteine-reactive fluorophores, which included versions of BODIPY fluorophores, Alexa dyes, CyDyes, 1-(3-(succinimidyloxycarbonyl) benzyl)-4-(5-(4-methoxyphenyl) oxazol-2-yl)pyridinium bromide (PyMPO), and tetramethylrhodamine-maleimide (TMR).^26^ Only TMR-5-β_2_AR was found to combine sufficient photostability with a clearly detectable and specific change in fluorescence intensity upon agonist binding. Here, we employ another dye called BODIPY 493/503 and label β_2_AR with it at Cys265. This particular dye was not tested in the previous work,^26^ but it displayed analogous changes in environment sensitivity and β_2_AR conformation via the TMR label. We studied this receptor in the presence and absence of the full agonist BI-167107. Also, we performed controls that further support the suitability of BODIPY 493/503 as a conformational reporter of the β_2_AR at the SM level. In broad agreement with SM FRET experiments performed with a full-length minimal cysteine ß_2_AR mutant in detergent micelles,^19^ our results revealed that this protein fluctuates between several intensity states and that there is a clear shift in the equilibrium distribution of β_2_AR upon full agonist binding (in a lipid membrane environment). Our results showed that the dynamics of a receptor truncated at C365 β_2_AR-BODIPY 493/503, in vesicles without G-protein, happen at longer time scales than with a full-length minimal cysteine β_2_AR-TMR mutant in detergent micelles.^18^ This agrees with β_2_AR-Cy3 (mutations E122W, C327S, and C341A, truncation beyond residue 348, and removal of residues 245–249) experiments performed in nanodiscs.^20^ In our work, ligand binding affected both the dwell times of the intensity states and the conformational pathways.

## Materials and Methods

### Preparation of Control Liposomes

Control liposomes with BODIPY FL-DHPE (Molecular Probes) conjugate and the lipid composition 1,2-dioleoyl-*sn*-glycero-3-phosphocholine (DOPC)/cholesteryl hemisuccinate (CHS)/1,2-dioleoyl-*sn*-glycero-3-phospho-*rac*-(1-glycerol) (DOPG)/1,2-dioleoyl-*sn*-glycero-3-phosphoethanolamine (DOPE)-ATTO655/1,2-distearoyl-*sn*-glycero-3-phosphoethanolamine (DSPE)-PEG(2000)-biotin (79.85:10:10:0.05:0.1) (Avanti polar lipids, Steraloids Inc., ATTO-Tec) were prepared by evaporating chloroform under argon and were dried 1 h under vacuum to prepare a thin lipid film. This control sample had 1 molecule of the BODIPY FL-1,2-dihexadecanoyl-*sn*-glycero-3-phosphoethanolamine (DHPE) per 10000 lipids. The film was resuspended in buffer (20 mM HEPES, 100 mM NaCl, pH 7.5) and vortexed for 5 min. Next, suspensions were freeze–thawed in 7 cycles of liquid nitrogen/37 °C water and extruded using 100 nm filters (Avanti polar lipids). These samples were aliquoted, frozen using liquid nitrogen, and stored until experiment at −80 °C.

### Protein Reconstitution into SUVs

A functional single reactive cysteine mutant of β_2_AR (β_2_AR–365-C265) was expressed, purified, and labeled with BODIPY 493/503 methyl bromide (BODIPY 493/503, 8-bromomethyl-4,4-difluoro-1,3,5,7-tetramethyl-4-bora-3a,4a-diaza-s-indacene, Invitrogen, B2103) as described previously.^27,28^ The β_2_AR protein construct was tagged N-terminally with the signal sequence MKTIIALSYIFCLVFA followed by FLAG epitope DYKDDDDA, the TEV protease recognition sequence ENLYFQGF, and the coding sequence of human β_2_AR encompassing Gly2 to Gly365. The truncation at 365 of β_2_AR removes two cysteines in the C-terminal tail that would otherwise have been labeled with BODIPY 493/503. An N-linked glycosylation site in second extracellular loop (ECL2) was removed by mutation of Asn187 to Glu.^27^ The β_2_AR receptor was solubilized according to methods described previously and purified using M1 anti-FLAG antibody chromatography (Sigma) prior to and after a purification by alprenolol-sepharose chromatography.^29^ We select for properly folded functional β_2_AR by the alprenolol-sepharose chromatography purification step, and therefore, the cysteines in ECL2 have intact disulfide bridges prior to exposure to thiol-reactive BODIPY 493/503. The receptor was labeled with 10 μM BODIPY 493/503 after the first M1 purification step in a receptor to a BODIPY 493/503 ratio of 1:6.6 for 45 min on ice. Any unlabeled available cysteines were quenched with 2 mM iodoacetamide. 100 μM TCEP was added to break up any potentially formed thiol-bridges between receptor proteins. Although the β_2_AR and its disulfide bridges tolerate the presence of 100 μM TCEP,^9^ the 200-fold higher concentration of iodoacetamide over the fluorophore ensures that any potential BODIPY 493/503 labeling of cysteines previously engaged in disulfide bridges is minimized to insignificance. Based on the absorption spectrum, we calculated that the molar ratio of BODIPY 493/503 incorporation is 0.15 mol BODIPY 493/503 to per mol purified β2AR-BODIPY 493/503 in DDM/CHS. The labeling is significantly lower than a 1:1 molar ratio, and therefore the likelihood of other cysteines besides Cys265 being labeled is low because Cys265 is the most solvent exposed cysteine in the construct. The other cysteines are more buried in the protein structure, palmitoylated, or form cysteine bridges.

Liposomes of the lipid composition DOPC/CHS/DOPG/DOPE-ATTO655/DSPE-PEG2000-biotin (79.85:10:10:0.05:0.1) (Avanti polar lipids, Steraloids Inc., ATTO-Tec) were prepared by evaporating chloroform under argon and were dried 1 h under vacuum to prepare a thin lipid film. The film was resuspended in buffer (20 mM HEPES, 100 mM NaCl, and 1% octylglucoside, pH 7.5), and the lipid-detergent mixture was formed by sonication for 1 h in an ice–water bath. Unlabeled β_2_AR was mixed with the labeled one to achieve a 1:10 labeled-to-unlabeled ratio. The lipid-detergent mixture and mixture of labeled and unlabeled β_2_AR were added in the 1:1000 protein-to-lipid ratio. The lipid-receptor mixture and sample buffer (20 mM HEPES, 100 mM NaCl, pH 7.5) (until 300 μL) were mixed and kept on ice for 2 h. Proteoliposomes were formed by the removal of detergent on a Sephadex G-50 (fine) column (25 cm × 0.8 cm). These samples were aliquoted, frozen using liquid nitrogen, and stored until experiment at −80 °C.

### Single Proteoliposome Immobilization

Proteoliposomes were immobilized on a passivated glass surface in home-built chambers and imaged by TIRF microscopy. Chamber parts were cleaned extensively by using ethanol and Milli-Q water (MQ; Millipore). Glass slides (thickness 170 ± 10 μm) were cleaned by consecutive rounds of sonication by 2% (v/v) Helmanex following three washes (×3) with MQ and ×2 with methanol. Glass slides were dried in nitrogen flow, plasma etched for 2 min, mounted in a microscope chamber, and incubated with a mixture of 1000:6 PLL-*g*-PEG and PLL-*g*-PEG-biotin (SuSoS, Switzerland) (1 g/L) in surface buffer (15 mM HEPES, pH 5.6) for 30 min. After carefully washing with a sample buffer (20 mM HEPES, 100 mM NaCl, pH 7.5), we incubated the surfaces with 0.1 g/L neutravidin (Life Technologies) in the surface buffer for 10 min after additional washing with sample buffer. Proteoliposome surface density was controlled by addition of 4 μL (0.05 g/L) proteoliposomes to an 80 μL chamber volume. The chamber was washed × 10 times in a sample buffer when the desired surface density reached. Before imaging, sample buffer containing 2.5 mM protocathechuic acid (PCA), 50 nM protocatechute-3,4-dioxygenase (PCD), and 1 mM 6-hydroxy-2,5,7,8-tetramethylchroman-2-carboxylic acid (Trolox) was injected into the chamber.

### TIRF Microscopy

Fluorescence images were acquired on an ECLIPSE Ti-E epifluoresence/TIRF microscope (NIKON, Japan) equipped with 405, 488, 561, and 647 nm lasers (Coherent, California). All lasers are individually shuttered and collected in a single fiber to the sample through a 1.49 NA, ×100, apochromat TIRF oil objective (NIKON, Japan). A dichroic mirror for the 488 nm laser (ZT491rdcxt, CHROMA) was used. The excitation light was filtered using a laser clean up filter (ZET 488/10x, CHROMA). The emitted light was filtered using a band-pass filter (ET bandpass 532/50, CHROMA). Images were recorded with an EM-CCD camera (iXon3 897, Andor). To keep the sample in focus over time, we employed a perfect focusing system (NIKON, Japan). Exposure time of the EM-CCD camera was set to 400 ms. We sampled at various rates but did not observe different dynamics below a 400 ms exposure. Such an exposure time also allowed us to collect signals of sufficient quality with the chosen laser excitation power density. The laser power was optimized to achieve the best possible signal-to-noise ratio and long enough bleaching time for the BODIPY fluorophore.

### SM Data Analysis

As previously described, all data analysis procedures were performed and graphs were prepared in the Igor Pro 6.37 (Wavemetrics) program using a custom-written analysis package (available upon direct request to the author).^30^ Briefly, SM signals (intensity versus time traces) were extracted from automatically detected fluorescent spots using a 2D Gaussian fitting with the center position and width kept constant. The intensity was expressed as a Gaussian integral. SM signals were selected, and multiple molecules containing signals were rejected manually. Also, signals that did not bleach completely until the end of the trace were rejected. For the intensity change point (ICP) detection, minimal amplitude now was set to 1 standard deviation, and the sum of the duration of state had to be longer than a set value of 12 points. We only accessed states with durations longer than 12 frames since we could reliably detect them using automated detection algorithms that are unlimited in state number. The standard deviation factor for ICP analysis was determined for each trace individually by calculating the standard deviation of 10% piece from the end of the signal.

### Bulk Fluorescence Spectroscopy

Fluorescence emission spectra were recorded with a Horiba Jobin Yvon Fluoromax-4 spectrometer. For all spectra, the same Hellma 45 μL microcuvette was used, slit width was kept constant at 5 nm, and excitation was set at 488 nm wavelength. During the experiment, the sample was injected into a cuvette, its average fluorescence spectrum was measured (three scans averaged), then the full-agonist BI-167107 was injected, and again an average fluorescence spectrum was acquired after 40 min of incubation time. The fluorescence intensity was calculated by taking the integral of the average spectrum. The change of fluorescence intensity upon the full agonist injection was then calculated by subtracting the intensity with the full agonist by the intensity without it.

## Results and Discussion

### Labeling β_2_AR with a Single Fluorophore for Conformational Dynamics Studies

**Figure 1:**
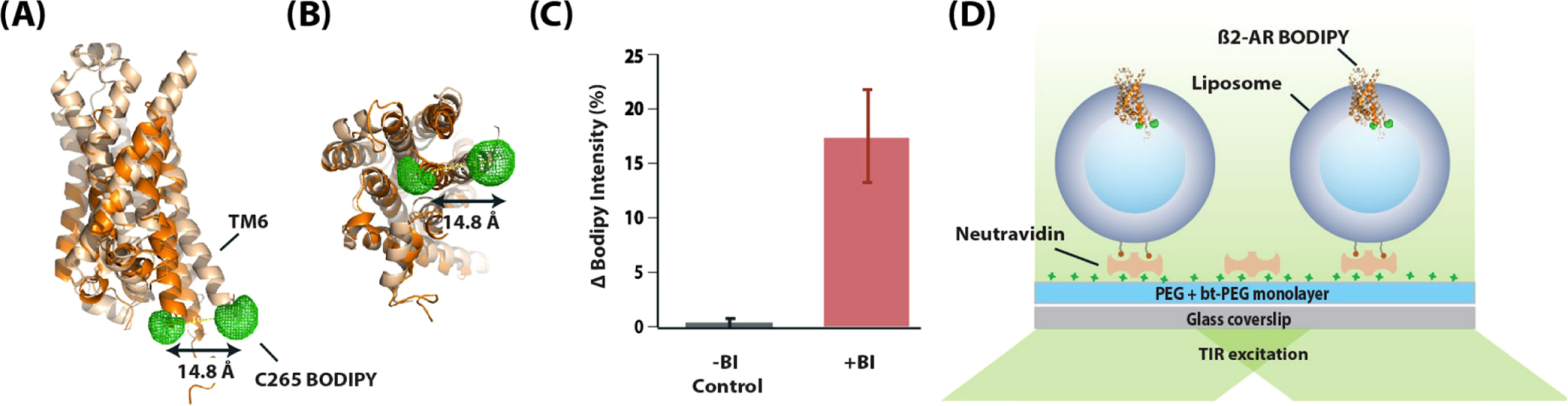
Labeling of β_2_AR with the BODIPY 493/503 fluorophore, ensemble spectroscopy results, and the assay of immobilized β_2_AR-BODIPY 493/503 proteoliposomes. (A) Side view of β_2_AR overlaid Apo (2R4S PDB ID, orange) and full agonist BI-167107 stabilized (4LDE PDB ID, wheat) crystal structures with simulated accessible volume (AV) clouds for BODIPY 493/503 at the C265 position. Distance change of AV centers between Apo and full agonist stabilized form is ∼15 Å. (B) Bottom view of crystal structures shown in panel (A). (C) Results of the receptor in proteoliposome activation measured in bulk by fluorescence spectroscopy. After 40 min incubation with the full agonist, we observed an increase in BODIPY 493/503 intensity by ∼17%. (D) TIRF microscopy assay to monitor SM conformational dynamics of β_2_AR-BODIPY 493/503 in liposomes. Liposomes contained 0.05% ATTO655-lipid conjugates which allowed for colocalization with the receptor. Liposomes also contained biotinylated lipids, which allowed for immobilization on glass surfaces functionalized with PLL–PEG/PLL–PEG-Biotin/neutravidin.

Upon activation by an agonist, the TM6 of β_2_AR moves toward the plasma membrane (Figure 1A,B).^9^ According to an accessible volume clouds simulation,^31^ this movement should be ∼1.5 nm. Upon a conformation change from the closed to opened state, the intracellular end of TM6 experiences a change in the polarity of its environment. The inside of the intracellular β_2_AR surface contains tyrosine (Tyr) residues (in the inactive state of β_2_AR, Tyr141 of ICL2 is closer to Cys265 (∼10 to 15 Å) compared to the active state, where TM6 moves away from TM3 and ICL2 curls up into helix further displacing Ty141) that can quench fluorophores.^32−34^ Thus, labeling the cytoplasmic end of the TM6 with a fluorophore sensitive to those factors can monitor fluorescence changes related to the conformational dynamics of the protein. Similar approaches have been applied to study activation of this protein in bulk with several different fluorophores or with a fluorophore-quencher pair.^26,35,36^

In this work, we labeled β_2_AR with the BODIPY 493/503 fluorophore. It was demonstrated that BODIPY FL, TMR, and other dyes are quenched by the Tyr residue,^32,34^ but the size of BODIPY fluorophores compared to TMR (and its distance sensitivity to the Tyr residue) makes it more favorable for SM imaging.^37^ It was suggested that the brightness of BODIPY 493/503 increases upon the change in environment from water to lipids, but its sensitivity to neither Trp nor Tyr was tested.^38^ We tested the effect of different solvents and Tyr and Trp to this dye using absorption, fluorescence emission, and fluorescence lifetime spectroscopy. These experiments confirmed that polarity is indeed important, and BODIPY 493/503 is sensitive to the polarity of the environment (SI Figure 1A,D,G). Its absorption spectrum is really sensitive to environmental polarity, shifts toward the shorter wavelengths, and becomes wider upon change in polarity from cyclohexane (CHX) to water (SI Figure 1A). Its fluorescence spectrum peak position was only marginally affected by the change in polarity, while fluorescence intensity dropped down upon the polarity change caused by changing from CHX to water (SI Figure 1D). Also, in water, we observed a second fluorescence peak at ∼650 nm wavelength. Fluorescence decay kinetics were only marginally affected by the change in polarity, and slower kinetics for the red-peak were found in water (SI Figure 1G). In absorption spectra, we observed more clear Trp quenching of BODIPY 493/503, while Tyr had only a minor effect (SI Figure 1B,C). Indeed, at such a high μM concentration of Trp/Tyr, the average distance between the fluorophore and the quencher is >1.5 nm, and therefore, it is not sufficient to induce full quenching of BODIPY 493/503,^34^ but it is a good indicator for more detailed photophysics studies of this dye at the SM level.^39^

Therefore, we attached a BODIPY 493/503 fluorophore to the intracellular end of TM6 in a truncated single reactive cysteine mutant of β_2_AR (β_2_AR–365-C265) (Figure 1A,B). The missing part of the C-terminal tail does not significantly affect the dynamics and conformational changes of the TM domain as agonist-induced responses from bimane-labeled full-length β_2_AR (with 4 K.O. cystines incl. the two in C-term) and the truncated (at 365) β_2_AR look indistinguishable in detergent.^15,40^ It also behaves similarly in living cells: The binding affinities of such truncated β_2_AR for tritiated antagonist dihydroalprenolol (1.02 nM in insect cell membranes and 0.55 nM in nanodiscs, 1.05 nM purified receptor reconstituted in liposomes)^40,41^ and agonist isoproterenol (290 nM in insect cell membranes and 107.5 nM in nanodisc, 640 nM purified receptor reconstituted in liposomes)^40−42^ were measured previously. The dihydroalprenolol (0.66 nM in insect cell membranes) and isoproterenol (160 nM in insect cell membranes) affinities are similar to that of full-length versions of the β_2_AR where cysteines have been knocked-out.^43^ This suggests that the lipid environment surrounding the receptor has a larger effect on the receptor functional state than whether the C-terminal tail is full length or truncated by ∼40 a.a.

The labeled protein was reconstituted into vesicles composed from DOPC, DOPG, cholesterol, a low amount of biotinylated lipid, and a red fluorescent dye-lipid conjugate. Cholesterol and negatively charged lipids are important for the function of this GPCR, and DOPG is important for the structural stability of vesicles and for reducing the multilamellarity of vesicles. Orientation of receptors in such proteoliposome preparation was quantified previously and it contained a uniform receptor orientation (β_2_AR 90% outside out).^28^ We used a ratio of 1:10 000 labeled protein to lipid; the ratio of labeled to unlabeled protein was 1:10. This was important to achieve a higher amount of liposomes, with one fluorescently labeled receptor per liposome. Also, β_2_AR forms clusters,^44,45^ and we optimized our sample to have a single labeled receptor per cluster and per vesicle.

We tested the functionality of the β_2_AR proteoliposome sample at the ensemble level using fluorescence spectroscopy. This revealed a potent functional response to full-agonist BI-167107 binding. The intensity of BODIPY 493/503 fluorescence emission increased upon the full-agonist injection by roughly 17%, while the control showed no increase (Figure 1C). This fluorescence increase was comparable to the previously reported increase of β_2_AR-TMR upon agonist activation.^26^

### SM Fluorescence Microscopy of β_2_AR Proteoliposomes

To monitor conformational dynamics of β_2_AR-BODIPY in proteoliposomes, we employed TIRF microscopy and functional proteoliposome immobilization on the surface. The biotinylated lipid introduced into the lipid composition of the proteoliposomes allowed us to anchor proteoliposomes via neutravidin onto the PLL–PEG/PLL–PEG-biotin (10:1) modified glass coverslip surface (Figure 1D). As demonstrated previously, this immobilization strategy allows liposomes to remain intact.^46,47^ Labeling directly the proteoliposomes with a lipid-dye conjugate allowed us to colocalize receptor and liposome signals and thus exclude nonreconstituted receptors from further analysis. We were thus able to follow dynamics of TM6 for hundreds of single β_2_AR molecules reconstituted in the native-like environment of a lipid bilayer membrane in real time.

**Figure 2:**
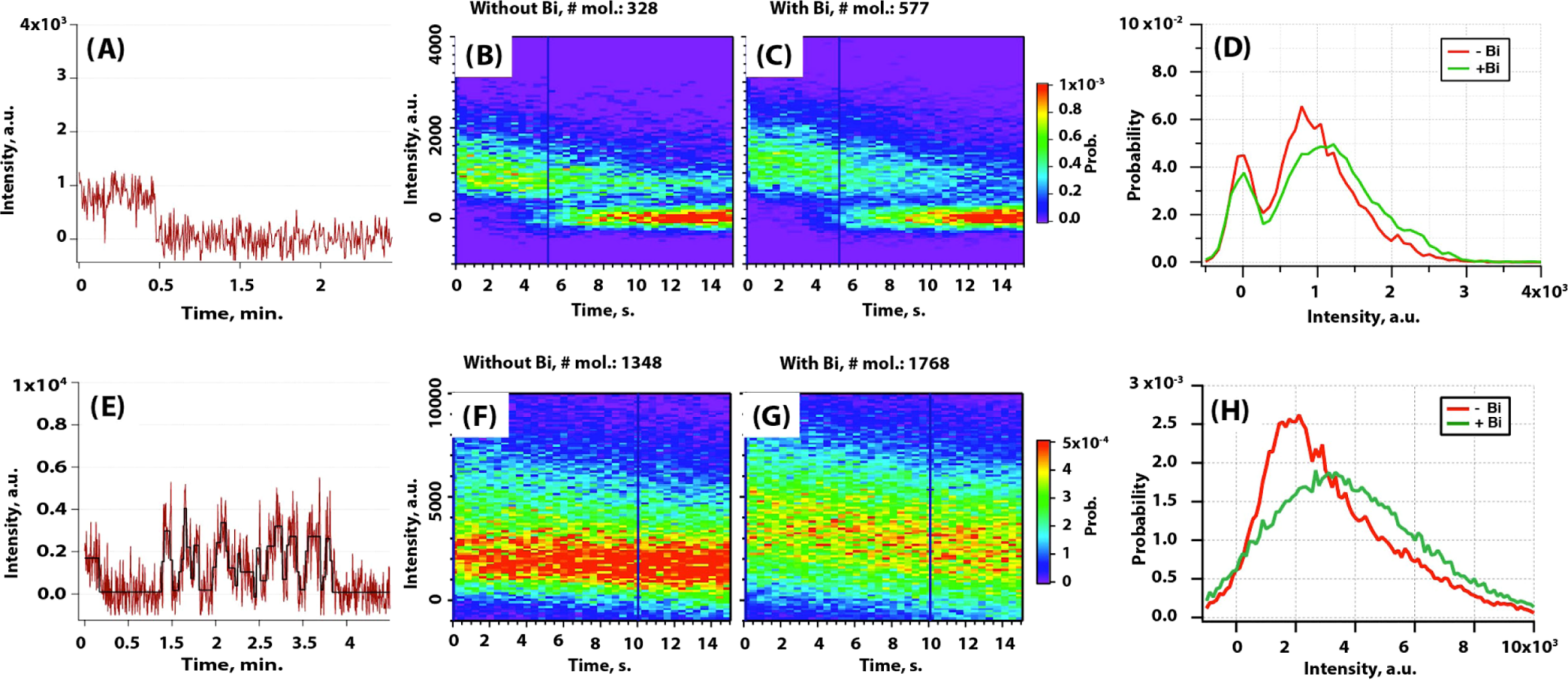
Single-molecule (SM) traces and SM emission population plots. (A) Representative SM trace of BODIPY-lipid conjugates in liposomes and (E) β_2_AR-BODIPY 493/503 in liposomes. Black lines indicate detected states by the automated intensity-change-point (ICP) detection algorithm. (B, C) SM population plots of BODIPY-lipid conjugates in liposomes and (F, G) β_2_AR-BODIPY in liposomes. (B, F) are in the absence of full agonist BI-167107, and (C, G) are in presence. (D, H) Vertical line scans of SM population plots from 0 until 5 s for BODIPY-lipid conjugates, and from 0 until 10 s for β_2_AR-BODIPY 493/503 conjugates. The number of molecules included in each plot is indicated on top of each plot, and plots are normalized to PDF. Color code indicated in the inset of plots represents probability.

Functionality of the sample and the fact that the BODIPY 493/503 fluorophore is quite stable motivated us to perform SM TIRF microscopy measurements. In these measurements, we acquired two channel images: green (BODIPY) and red (lipid-dye) channels. The green and red channel images were acquired respectively under excitation at 488 and 647 nm. An automated custom-written analysis procedure was used to perform detection of fluorescent spots in the acquired images and to check for their colocalization between these two channels. Only colocalizing spots that fitted well the 2D Gaussian function were taken into account. From these spots, by fitting 2D Gaussians to each frame in the image series, we extracted intensity over time traces (Figure 2A,E). These traces were manually inspected, and only those that fulfilled criteria for single-molecules were included into the plots presented below.

To represent ensemble average behavior of the SM population, we overlaid all selected SM traces onto SM population traces plots (Figure 2B,C,F,G). From these plots, we made vertical line-profiles that represented the distribution of intensities before bleaching occurs (Figure 2D,H). These plots showed two main peaks for the control liposome sample containing BODIPY-lipid: the first peak represents an emitting state with center at ∼800 au and the other–bleached state, which was centered at 0 au (Figure 2D). In contrast, for the β_2_AR-BODIPY 493/503 proteoliposomes, we observed a broader distribution of intensities that appeared to reflect the conformational transitions of the receptor (Figure 2H). The presence of the full-agonist slightly shifted the main fluorescent state of the control sample (∼400 au), but did not broaden it. This suggests that BI has an effect on BODIPY fluorescence. However, since β_2_AR in our proteoliposomes is oriented correctly (mainly outward-out),^28^ the BODIPY 493/503 dye always faces toward the inside of proteoliposomes, while never directly interacting with the ligand. Such unwanted photophysical effects on a reporter’s fluorescence emission are more problematic for nanodiscs and detergent micelle reconstituted β_2_AR samples.^18,20^

For the β_2_AR-BODIPY 493/503 proteoliposomes upon the full-agonist treatment, we observed redistribution of the states (Figure 2H). Probability of the moderately fluorescent states decreased while the probability of the higher fluorescent states increased. This result was similar to the previous studies with TMR-labeled β_2_AR in detergent micelles.^18^ However, previous work with β_2_AR-Cy3 in nanodiscs showed a decrease in intensity upon full agonist formoterol injection, which indicates different sensitivity of Cy3.^20^ Also, BODIPY 493/503 on β_2_AR in proteoliposomes, compared to BODIPY-lipid in control liposomes, became brighter and more photostable. This effect is likely caused by closer proximity to the hydrophobic environment once the BODIPY 493/503 is coupled to the protein. The bulk intensity change (Figure 1C) and the calculated average change (SI Figure 2) in intensity from SM signals (Figure 2H) shifted similarly (∼16%) to bulk spectroscopy.

### Conformational Dynamics of β_2_AR in Proteoliposomes

**Figure 3:**
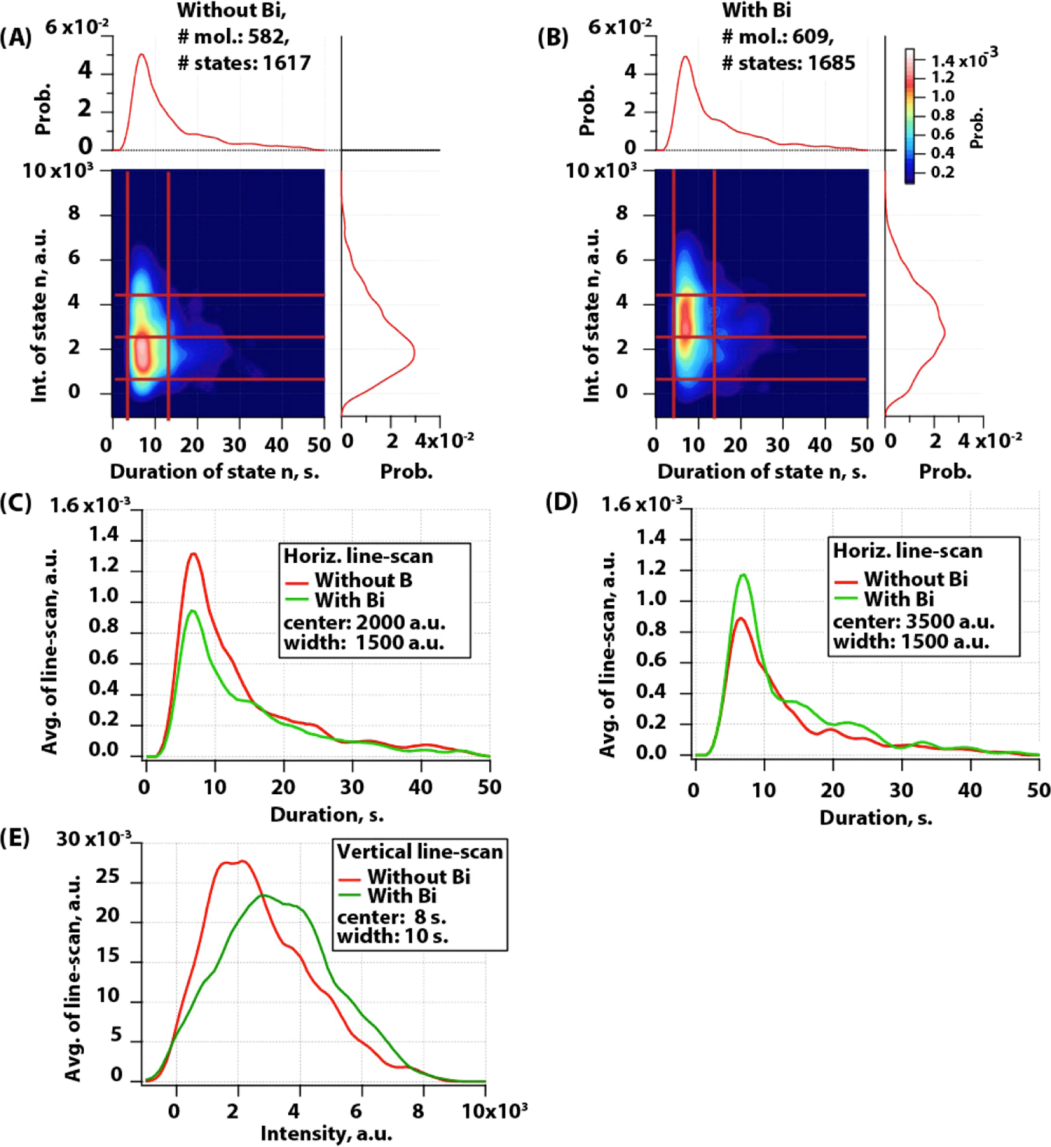
State duration 2D histogram plot of β_2_AR-BODIPY 493/503 in liposomes. (A) in the absence and (B) in the presence of the full agonist BI-167107. Plots normalized to PDF and color code represent probability. (C) Horizontal line scan of (A, B) for low fluorescence intensity states, and (D) high-intensity states. (E) Vertical line scan of (A, B) for states of dominating durations. Center and width of the line scan and color code are indicated in the legend of each graph.

To characterize the conformational dynamics of β_2_AR-BODIPY 493/503 in liposomes, we employed an automated intensity-change-point (ICP) detection algorithm that is not limited to a predefined number of states.^30,48^ This analysis algorithm allowed us to detect stably emitting states in the selected SM traces (Figure 2E) and provided their characteristics: state duration, average intensity, and transition paths. Information obtained by ICP detection was summarized using the 2D state duration histogram plot (Figure 3A,B). These plots represent duration of state n on the *x*-axis of the plot and average intensity of the corresponding state–on the *y*-axis. The color scale of these plots represents the probability to detect a given state at a certain point. The obtained plots showed dispersed distribution of state durations and intensities. The peak of distribution in the basal state compared to the full agonist bound was at lower intensities (1800 au compared to 3500 au), but roughly at the same state durations. This effect was present in the vertical line scan taken at shorter duration states (Figure 3E). We interpreted these findings as indications that binding of the full agonist shifts conformation of the protein toward the open-facing state but only marginally affects the duration of states.

To gain a more detailed view of state durations, we made two horizontal line scans of these 2D plots (Figure 3C,D). A horizontal line scan taken at lower intensity states showed that the probability of shorter duration states in the presence of the full agonist is lower than without it. In contrast, the line scan taken at higher intensity states showed that probability of practically all duration states in the presence of the full agonist is higher than without it (Figure 3D). That suggested that the full agonist causes stabilization of higher intensity states (becomes longer) at the expense of lowering the probability of the lower intensity states. However, the duration of states observed was not highly affected upon the full agonist binding. These conclusions are in qualitative agreement with the results of two previous studies.^18,19^

**Figure 4:**
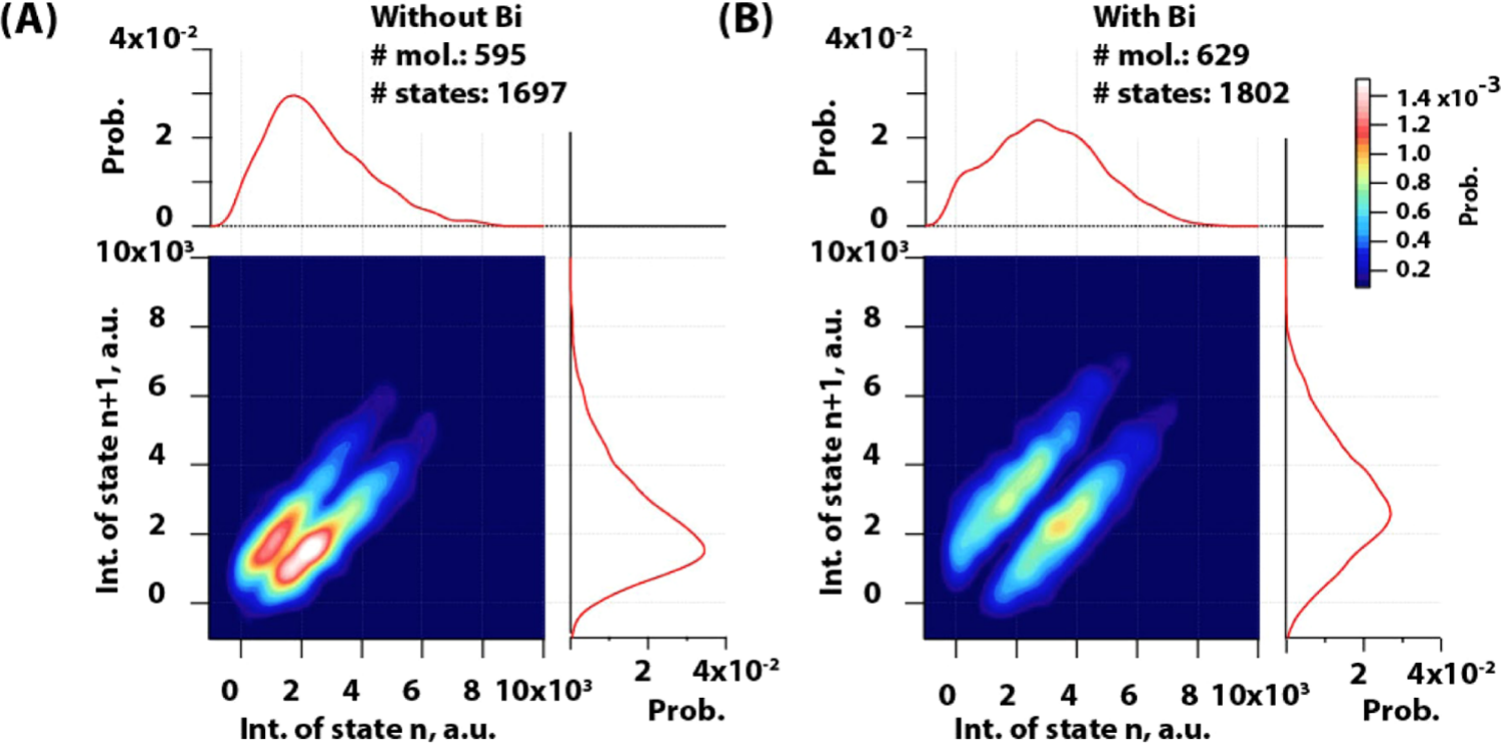
Transition density 2D histogram plot of β_2_AR-BODIPY 493/503 in liposomes. (A) in absence and (B) in the presence of the full agonist BI-167107. Plots normalized to PDF and color code represent probability.

To understand the conformational transitions of this protein, we made 2D transition density plots from information obtained by using the ICP algorithm (Figure 4). These plots represent an average intensity of state (*n*) at the *x*-axis of the plot and an average intensity of the following state that proteins transits to (*n* + 1) – on the *y*-axis. The color scale of these plots represents the probability to detect a given state. These plots showed a mirror view along the diagonal, and this indicated that the system is under equilibrium. In the absence of the full agonist, the receptor was mainly transiting between states of lower intensity (Figure 4A). This included transition from states having average intensity close to 2000 au into states 1500 au Only rarely protein was transiting in between states with higher intensity. In contrast, the full-agonist binding to the protein induced transition between higher intensity states. The most probable transition became from states of 3500 au into 2000 au The whole distribution showed that there are more than two states, but rather populations of substates that our probe is able to report. This was most evident in the absence of the full agonist, where the main peak appeared to be composed out of two populations: one representing transition from 1800 au into 1500 au and the other representing transition from 2300 au into 1800 au The low probability of transitions from completely quenched states into highly fluorescent states indicated that the receptor’s dominant transitions are affected and it rarely takes a transition from completely closed into fully opened conformation. Thus, ligand binding seems not to induce a pathway that is not sampled before, but it changes the most dominant transitions of this protein. However, differences between intensity and our signal-to-noise ratio did not allow us to discriminate these states in more detail.

A previous study of a full-length minimal cysteine β_2_AR-TMR (TMR at C265) mutant in a detergent environment, which was labeled at the same position of C265 but with a different environmentally sensitive dye–TMR, revealed a shift in dye intensity-lifetime space toward higher values upon the full agonist BI-167107 binding.^18^ This technique allowed the measurement of time scales of interconversion of fluctuating states that lasted from milliseconds to seconds. It also showed that the state dwell times increased from ∼130 to ∼200 ms upon full agonist binding.

The more recent study of a full-length minimal cysteine β_2_AR mutant, which was labeled at 266C and 148C positions with a FRET pair, in detergent micelles tracked movements of the TM6 in the presence of ligands with distinct efficacies and determined the effects on the receptor structure, dynamics, and G-protein coupling.^19^ This study revealed that partial and full agonists differentially affect TM6 fluctuations in two different ways: (1) the rate at which GDP-bound β_2_AR-Gs complexes are formed and (2) the efficiency of nucleotide exchange. Both of these actions lead to Gs activation. They also showed insights of transient nucleotide-bound β_2_AR-Gs species that are distinct from known structures and provided potential insights into the allosteric link between ligand-and nucleotide-binding pockets. Without G-protein FRET trajectories revealed only rare fluctuations (around 1 min dwell time), but correlation analyses revealed clear signatures of fast (∼10 ms dwell time, which is below the time resolution of 100 ms that they have used in all experiments) reversible TM6 movements. By this measure, more rapid TM6 dynamics were observed in agonist-bound samples than antagonist-bound samples.

The study of lipid nanodisc-reconstituted β_2_AR-Cy3 (Cy3 at Cys265, contained E122W, C327S, and C341A mutations, was truncated at residue 348, and residues 245–249 were removed) revealed that this receptor spontaneously transits between two distinct conformational states (inactive and active) and their dwell time was in the range of 0.2–2 s.^20^ In the apo form, the receptor was sampling both conformations with a bias toward the inactive state. Binding of the full-agonist formoterol shifted conformational distribution toward the active-like conformation, whereas binding of the inverse agonist ISI-118,551 favored the inactive conformation. Also, binding of formoterol increased the frequency of activation transitions at the expense of reduced frequency of deactivation transition events. In contrast, the inverse agonist increased the frequency of deactivation transitions.

In comparison to this, our study with vesicle-reconstituted β_2_AR-BODIPY 493/503, which was truncated at C365 and labeled at the end of TM6 on residue C265 with BODIPY 493/503, characterized dwell times of different average intensity states that lasted from long milliseconds to minutes. Our results, as well as the aforementioned TMR-based study of the full-length minimal cysteine β_2_AR mutant in detergent micelles, showed that probability and dwell time of the higher intensity states increased. However, our study revealed slower dynamics of TM6 and due to longer observation times enables tracking of the dominant conformational transition. This helped us suggest that interconversion between states of higher intensity is more probable upon the full agonist binding.

In agreement with the aforementioned Cy3-labeled β_2_AR studies in lipid nanodiscs,^20^ our results suggested that the receptor fluctuates between several states of different intensity and that there is a clear shift in the equilibrium distribution of β_2_AR upon full agonist binding in a lipid membrane environment. In the β_2_-AR-Cy3 study, Cy3 intensity decreased upon full agonist injection, while in our study and β_2_-AR-TMR study, intensity of the reporter increased upon the full agonist injection. That demonstrates Cy3 sensitivity to different factors compared to the environment polarity. Additionally, in our study, ligand binding appeared to have an effect on the dwell times of states and the dominant transitions.

## Conclusions

In this work, we correlated activities of the β_2_AR with time-dependent changes in the fluorescence intensity of an environmentally sensitive BODIPY 493/503 fluorophore attached to the native cysteine residue found at the intracellular end of transmembrane helix 6 (TM6) of β_2_AR. Intensity changes in BODIPY 493/503 fluorescence report on changes in the TM6 position that accompany β_2_AR activation. Associations of this kind have been made for other environmentally sensitive fluorophores linked to TM6, such as bimane and TMR,^8,40^ where the movement of TM6 alters the local quenching phenomenon of the attached fluorophore to change its effective rate of photon emission. However, specifically for BODIPY 493/503 environmental sensitivity was not studied yet, and here we demonstrated that its absorbance is sensitive to polarity and Trp/Tyr, while fluorescence is only marginally sensitive to those factors under our experimental conditions. This SM level study of β_2_AR conformational dynamics was conducted in lipid vesicles, which is a native-like environment. Surface tethering of fluorescently labeled proteoliposomes allowed us to perform long-lasting stable observations of hundreds of SM β_2_AR receptors in parallel. Lipid-dye and BODIPY 493/503 colocalization helped us easily select only liposome-reconstituted preceptors and discard nonreconstituted proteins from the analysis. Our results show that β_2_AR-BODIPY fluctuates between several states of different intensity on a time scale of seconds, while BODIPY-lipid conjugates have rather stable fluorescence emission in the absence and presence of the full agonist BI-167107. Full agonist stimulation changes the β_2_AR dynamics, increasing the population of states with higher intensities and slightly prolonging their duration (consistent with bulk experiments). In addition, our data suggest that in the absence of the full agonist, β_2_AR-BODIPY interconverts between states of low and moderate intensity, while the full agonist induces change in the conformational distribution of the receptor so that transitions between the moderate and high-intensity states become more probable. This redistribution is consistent with a mechanism of conformational selection. Results of ligand binding affinity studies of β_2_AR in detergent, nanodiscs, vesicles, and living cells^40−43^ and comparison of dwell times in previous SM conformational dynamics reports done in either detergent micelles^18,19^ or nanodiscs^20^ together with our results in vesicles suggest that the lipid membrane plays a role in slowing down TM6 conformational transitions of β_2_AR. Capturing β_2_AR conformational dynamics at the SM level in a lipid bilayer is a promising first step toward characterizing conformational dynamics of GPCRs in more complex native cellular membranes.

## Supporting Information Available

Materials and methods description of absorption, steady-state, and time-resolved fluorescence. Results of absorbance and fluorescence emission spectra, and time-resolved fluorescence decays of BODIPY 493/503, and their sensitivity to polarity and tryptophan or tyrosine. Vertical line scans of single-molecule population plots from 0 until 10 s for β_2_AR-BODIPY 493/503 conjugates (PDF)

## References

[ref1] Pierce K. L.; Premont R. T.; Lefkowitz R. J. Seven-Transmembrane Receptors. Nat. Rev. Mol. Cell Biol. 2002, 3 (9), 639–650. 10.1038/nrm908. 12209124

[ref2] Vilardaga J.-P.; Bünemann M.; Feinstein T. N.; Lambert N.; Nikolaev V. O.; Engelhardt S.; Lohse M. J.; Hoffmann C. GPCR and G Proteins: Drug Efficacy and Activation in Live Cells. Mol. Endocrinol. 2009, 23 (5), 590–599. 10.1210/me.2008-0204. 19196832 PMC5419261

[ref3] Kenakin T. New Concepts in Pharmacological Efficacy at 7TM Receptors: IUPHAR Review 2. Br. J. Pharmacol. 2013, 168 (3), 554–575. 10.1111/j.1476-5381.2012.02223.x. 22994528 PMC3579279

[ref4] Kenakin T. P. Cellular Assays as Portals to Seven-Transmembrane Receptor-Based Drug Discovery. Nat. Rev. Drug Discovery 2009, 8 (8), 617–626. 10.1038/nrd2838. 19609267

[ref5] Manglik A.; Kobilka B. The Role of Protein Dynamics in GPCR Function: Insights from the β2AR and Rhodopsin. Curr. Opin. Cell Biol. 2014, 27, 136–143. 10.1016/j.ceb.2014.01.008. 24534489 PMC3986065

[ref6] Frauenfelder H.; Parak F.; Young R. D. Conformational Substates in Proteins. Annu. Rev. Biophys. Biophys. Chem. 1988, 17, 451–479. 10.1146/annurev.bb.17.060188.002315. 3293595

[ref7] Baker J. G. The Selectivity of Beta-Adrenoceptor Agonists at Human beta1-, beta2- and beta3-Adrenoceptors. Br. J. Pharmacol. 2010, 160 (5), 1048–1061. 10.1111/j.1476-5381.2010.00754.x. 20590599 PMC2936015

[ref8] Swaminath G.; Xiang Y.; Lee T. W.; Steenhuis J.; Parnot C.; Kobilka B. K. Sequential Binding of Agonists to the β2 Adrenoceptor. J. Biol. Chem. 2004, 279 (1), 686–691. 10.1074/jbc.M310888200. 14559905

[ref9] Rasmussen S. G. F.; DeVree B. T.; Zou Y.; Kruse A. C.; Chung K. Y.; Kobilka T. S.; Thian F. S.; Chae P. S.; Pardon E.; Calinski D.; et al. Crystal Structure of the β2 Adrenergic Receptor-Gs Protein Complex. Nature 2011, 477 (7366), 549–555. 10.1038/nature10361. 21772288 PMC3184188

[ref10] Kruse A. C.; Ring A. M.; Manglik A.; Hu J.; Hu K.; Eitel K.; Hübner H.; Pardon E.; Valant C.; Sexton P. M.; et al. Activation and Allosteric Modulation of a Muscarinic Acetylcholine Receptor. Nature 2013, 504 (7478), 101–106. 10.1038/nature12735. 24256733 PMC4020789

[ref11] Huang W.; Manglik A.; Venkatakrishnan A. J.; Laeremans T.; Feinberg E. N.; Sanborn A. L.; Kato H. E.; Livingston K. E.; Thorsen T. S.; Kling R. C.; et al. Structural Insights into μ-Opioid Receptor Activation. Nature 2015, 524 (7565), 315–321. 10.1038/nature14886. 26245379 PMC4639397

[ref12] Carpenter B.; Nehmé R.; Warne T.; Leslie A. G. W.; Tate C. G. Structure of the Adenosine A(2A) Receptor Bound to an Engineered G Protein. Nature 2016, 536 (7614), 104–107. 10.1038/nature18966. 27462812 PMC4979997

[ref13] Yao X. J.; Vélez Ruiz G.; Whorton M. R.; Rasmussen S. G. F.; DeVree B. T.; Deupi X.; Sunahara R. K.; Kobilka B. The Effect of Ligand Efficacy on the Formation and Stability of a GPCR-G Protein Complex. Proc. Natl. Acad. Sci. U. S. A. 2009, 106 (23), 9501–9506. 10.1073/pnas.0811437106. 19470481 PMC2685739

[ref14] Manglik A.; Kim T. H.; Masureel M.; Altenbach C.; Yang Z.; Hilger D.; Lerch M. T.; Kobilka T. S.; Thian F. S.; Hubbell W. L.; et al. Structural Insights into the Dynamic Process of β2-Adrenergic Receptor Signaling. Cell 2015, 161 (5), 1101–1111. 10.1016/j.cell.2015.04.043. 25981665 PMC4441853

[ref15] Nygaard R.; Zou Y.; Dror R. O.; Mildorf T. J.; Arlow D. H.; Manglik A.; Pan A. C.; Liu C. W.; Fung J. J.; Bokoch M. P.; et al. The Dynamic Process of β(2)-Adrenergic Receptor Activation. Cell 2013, 152 (3), 532–542. 10.1016/j.cell.2013.01.008. 23374348 PMC3586676

[ref16] Rosenbaum D. M.; Zhang C.; Lyons J. A.; Holl R.; Aragao D.; Arlow D. H.; Rasmussen S. G. F.; Choi H.-J.; Devree B. T.; Sunahara R. K.; et al. Structure and Function of an Irreversible Agonist-β(2) Adrenoceptor Complex. Nature 2011, 469 (7329), 236–240. 10.1038/nature09665. 21228876 PMC3074335

[ref17] Peleg G.; Ghanouni P.; Kobilka B. K.; Zare R. N. Single-Molecule Spectroscopy of the beta(2) Adrenergic Receptor: Observation of Conformational Substates in a Membrane Protein. Proc. Natl. Acad. Sci. U. S. A. 2001, 98 (15), 8469–8474. 10.1073/pnas.151239698. 11438704 PMC37459

[ref18] Bockenhauer S.; Fürstenberg A.; Yao X. J.; Kobilka B. K.; Moerner W. E. Conformational Dynamics of Single G Protein-Coupled Receptors in Solution. J. Phys. Chem. B 2011, 115 (45), 13328–13338. 10.1021/jp204843r. 21928818 PMC3213290

[ref19] Gregorio G. G.; Masureel M.; Hilger D.; Terry D. S.; Juette M.; Zhao H.; Zhou Z.; Perez-Aguilar J. M.; Hauge M.; Mathiasen S.; et al. Single-Molecule Analysis of Ligand Efficacy in β2AR-G-Protein Activation. Nature 2017, 547 (7661), 68–73. 10.1038/nature22354. 28607487 PMC5502743

[ref20] Lamichhane R.; Liu J. J.; Pljevaljcic G.; White K. L.; van der Schans E.; Katritch V.; Stevens R. C.; Wüthrich K.; Millar D. P. Single-Molecule View of Basal Activity and Activation Mechanisms of the G Protein-Coupled Receptor β2AR. Proc. Natl. Acad. Sci. U. S. A. 2015, 112 (46), 14254–14259. 10.1073/pnas.1519626112. 26578769 PMC4655547

[ref21] Lamichhane R.; Liu J. J.; White K. L.; Katritch V.; Stevens R. C.; Wüthrich K.; Millar D. P. Biased Signaling of the G-Protein-Coupled Receptor β2AR Is Governed by Conformational Exchange Kinetics. Structure 2020, 28 (3), 371–377.e3. 10.1016/j.str.2020.01.001. 31978323 PMC7213800

[ref22] Lohse M. J.; Nikolaev V. O.; Hein P.; Hoffmann C.; Vilardaga J.-P.; Bünemann M. Optical Techniques to Analyze Real-Time Activation and Signaling of G-Protein-Coupled Receptors. Trends Pharmacol. Sci. 2008, 29 (3), 159–165. 10.1016/j.tips.2007.12.002. 18262662

[ref23] Larsen J. B.; Jensen M. B.; Bhatia V. K.; Pedersen S. L.; Bjørnholm T.; Iversen L.; Uline M.; Szleifer I.; Jensen K. J.; Hatzakis N. S.; et al. Membrane Curvature Enables N-Ras Lipid Anchor Sorting to Liquid-Ordered Membrane Phases. Nat. Chem. Biol. 2015, 11 (3), 192–194. 10.1038/nchembio.1733. 25622090

[ref24] Hatzakis N. S.; Bhatia V. K.; Larsen J.; Madsen K. L.; Bolinger P.-Y.; Kunding A. H.; Castillo J.; Gether U.; Hedegård P.; Stamou D. How Curved Membranes Recruit Amphipathic Helices and Protein Anchoring Motifs. Nat. Chem. Biol. 2009, 5 (11), 835–841. 10.1038/nchembio.213. 19749743

[ref25] Iversen L.; Mathiasen S.; Larsen J. B.; Stamou D. Membrane Curvature Bends the Laws of Physics and Chemistry. Nat. Chem. Biol. 2015, 11 (11), 822–825. 10.1038/nchembio.1941. 26485070

[ref26] Neumann L.; Wohland T.; Whelan R. J.; Zare R. N.; Kobilka B. K. Functional Immobilization of a Ligand-Activated G-Protein-Coupled Receptor. ChemBioChem 2002, 3 (10), 993–998. 10.1002/1439-7633(20021004)3:103.0.CO;2-Y. 12362365

[ref27] Rasmussen S. G. F.; Choi H.-J.; Rosenbaum D. M.; Kobilka T. S.; Thian F. S.; Edwards P. C.; Burghammer M.; Ratnala V. R. P.; Sanishvili R.; Fischetti R. F.; et al. Crystal Structure of the Human beta2 Adrenergic G-Protein-Coupled Receptor. Nature 2007, 450 (7168), 383–387. 10.1038/nature06325. 17952055

[ref28] Fung J. J.; Deupi X.; Pardo L.; Yao X. J.; Velez-Ruiz G. A.; Devree B. T.; Sunahara R. K.; Kobilka B. K. Ligand-Regulated Oligomerization of beta(2)-Adrenoceptors in a Model Lipid Bilayer. EMBO J. 2009, 28 (21), 3315–3328. 10.1038/emboj.2009.267. 19763081 PMC2748299

[ref29] Kobilka B. K. Amino and Carboxyl Terminal Modifications to Facilitate the Production and Purification of a G Protein-Coupled Receptor. Anal. Biochem. 1995, 231 (1), 269–271. 10.1006/abio.1995.1533. 8678314

[ref30] Tutkus M.; Chmeliov J.; Rutkauskas D.; Ruban A. V.; Valkunas L. Influence of the Carotenoid Composition on the Conformational Dynamics of Photosynthetic Light-Harvesting Complexes. J. Phys. Chem. Lett. 2017, 8 (23), 5898–5906. 10.1021/acs.jpclett.7b02634. 29140702

[ref31] Kalinin S.; Peulen T.; Sindbert S.; Rothwell P. J.; Berger S.; Restle T.; Goody R. S.; Gohlke H.; Seidel C. A. M. A Toolkit and Benchmark Study for FRET-Restrained High-Precision Structural Modeling. Nat. Methods 2012, 9 (12), 1218–1225. 10.1038/nmeth.2222. 23142871

[ref32] Mansoor S. E.; McHaourab H. S.; Farrens D. L. Mapping Proximity within Proteins Using Fluorescence Spectroscopy. A Study of T4 Lysozyme Showing That Tryptophan Residues Quench Bimane Fluorescence. Biochemistry 2002, 41 (8), 2475–2484. 10.1021/bi011198i. 11851393

[ref33] Doose S.; Neuweiler H.; Sauer M. A Close Look at Fluorescence Quenching of Organic Dyes by Tryptophan. ChemPhysChem 2005, 6 (11), 2277–2285. 10.1002/cphc.200500191. 16224752

[ref34] Jones Brunette A. M.; Farrens D. L. Distance Mapping in Proteins Using Fluorescence Spectroscopy: Tyrosine, like Tryptophan, Quenches Bimane Fluorescence in a Distance-Dependent Manner. Biochemistry 2014, 53 (40), 6290–6301. 10.1021/bi500493r. 25144569 PMC4196733

[ref35] Yao X.; Parnot C.; Deupi X.; Ratnala V. R. P.; Swaminath G.; Farrens D.; Kobilka B. Coupling Ligand Structure to Specific Conformational Switches in the beta2-Adrenoceptor. Nat. Chem. Biol. 2006, 2 (8), 417–422. 10.1038/nchembio801. 16799554

[ref36] Ghanouni P.; Steenhuis J. J.; Farrens D. L.; Kobilka B. K. Agonist-Induced Conformational Changes in the G-Protein-Coupling Domain of the Beta 2 Adrenergic Receptor. Proc. Natl. Acad. Sci. U. S. A. 2001, 98 (11), 5997–6002. 10.1073/pnas.101126198. 11353823 PMC33412

[ref37] Marmé N.; Knemeyer J.-P.; Sauer M.; Wolfrum J. Inter- and Intramolecular Fluorescence Quenching of Organic Dyes by Tryptophan. Bioconjugate Chem. 2003, 14 (6), 1133–1139. 10.1021/bc0341324. 14624626

[ref38] Chen J.; Liu W.; Fang X.; Qiao Q.; Xu Z. BODIPY 493 Acts as a Bright Buffering Fluorogenic Probe for Super-Resolution Imaging of Lipid Droplet Dynamics. Chin. Chem. Lett. 2022, 33 (12), 5042–5046. 10.1016/j.cclet.2022.03.120.

[ref39] Smit J. H.; van der Velde J. H. M.; Huang J.; Trauschke V.; Henrikus S. S.; Chen S.; Eleftheriadis N.; Warszawik E. M.; Herrmann A.; Cordes T. On the Impact of Competing Intra- and Intermolecular Triplet-State Quenching on Photobleaching and Photoswitching Kinetics of Organic Fluorophores. Phys. Chem. Chem. Phys. 2019, 21 (7), 3721–3733. 10.1039/C8CP05063E. 30499568

[ref40] Rasmussen S. G. F.; Choi H.-J.; Fung J. J.; Pardon E.; Casarosa P.; Chae P. S.; Devree B. T.; Rosenbaum D. M.; Thian F. S.; Kobilka T. S.; et al. Structure of a Nanobody-Stabilized Active State of the β(2) Adrenoceptor. Nature 2011, 469 (7329), 175–180. 10.1038/nature09648. 21228869 PMC3058308

[ref41] Bokoch M. P.; Zou Y.; Rasmussen S. G. F.; Liu C. W.; Nygaard R.; Rosenbaum D. M.; Fung J. J.; Choi H.-J.; Thian F. S.; Kobilka T. S.; et al. Ligand-Specific Regulation of the Extracellular Surface of a G-Protein-Coupled Receptor. Nature 2010, 463 (7277), 108–112. 10.1038/nature08650. 20054398 PMC2805469

[ref42] Day P. W.; Rasmussen S. G. F.; Parnot C.; Fung J. J.; Masood A.; Kobilka T. S.; Yao X.-J.; Choi H.-J.; Weis W. I.; Rohrer D. K.; et al. A Monoclonal Antibody for G Protein–coupled Receptor Crystallography. Nat. Methods 2007, 4 (11), 927–929. 10.1038/nmeth1112. 17952087

[ref43] Jensen A. D.; Guarnieri F.; Rasmussen S. G.; Asmar F.; Ballesteros J. A.; Gether U. Agonist-Induced Conformational Changes at the Cytoplasmic Side of Transmembrane Segment 6 in the Beta 2 Adrenergic Receptor Mapped by Site-Selective Fluorescent Labeling. J. Biol. Chem. 2001, 276 (12), 9279–9290. 10.1074/jbc.M004871200. 11118431

[ref44] Calebiro D.; Rieken F.; Wagner J.; Sungkaworn T.; Zabel U.; Borzi A.; Cocucci E.; Zürn A.; Lohse M. J. Single-Molecule Analysis of Fluorescently Labeled G-Protein-Coupled Receptors Reveals Complexes with Distinct Dynamics and Organization. Proc. Natl. Acad. Sci. U. S. A. 2013, 110 (2), 743–748. 10.1073/pnas.1205798110. 23267088 PMC3545784

[ref45] Mathiasen S.; Christensen S. M.; Fung J. J.; Rasmussen S. G. F.; Fay J. F.; Jorgensen S. K.; Veshaguri S.; Farrens D. L.; Kiskowski M.; Kobilka B.; et al. Nanoscale High-Content Analysis Using Compositional Heterogeneities of Single Proteoliposomes. Nat. Methods 2014, 11 (9), 931–934. 10.1038/nmeth.3062. 25086504 PMC4485457

[ref46] Bendix P. M.; Pedersen M. S.; Stamou D. Quantification of Nano-Scale Intermembrane Contact Areas by Using Fluorescence Resonance Energy Transfer. Proc. Natl. Acad. Sci. U. S. A. 2009, 106 (30), 12341–12346. 10.1073/pnas.0903052106. 19597158 PMC2709668

[ref47] Sarmento M. J.; Prieto M.; Fernandes F. Reorganization of Lipid Domain Distribution in Giant Unilamellar Vesicles upon Immobilization with Different Membrane Tethers. Biochim. Biophys. Acta 2012, 1818 (11), 2605–2615. 10.1016/j.bbamem.2012.05.028. 22664063

[ref48] Krüger T. P. J.; Ilioaia C.; van Grondelle R. Fluorescence Intermittency from the Main Plant Light-Harvesting Complex: Resolving Shifts between Intensity Levels. J. Phys. Chem. B 2011, 115 (18), 5071–5082. 10.1021/jp201609c. 21452800

