# Supplementary material for: Probing Activation and Conformational Dynamics of the Vesicle-Reconstituted β2 Adrenergic Receptor at the Single-Molecule Level
Source: J Phys Chem B. 2024 Feb 23;128(9):2124–33. doi: 10.1021/acs.jpcb.3c08349 (PMC10926102; doi:10.1021/acs.jpcb.3c08349)
Supplement: Supplementary file 1 — jp3c08349_si_001.pdf [file jp3c08349_si_001.pdf]

Supporting Information for Publication:

Probing activation and conformational dynamics of the vesicle-reconstituted ß2 adrenergic receptor at the single-molecule level

Marijonas Tutkus [1,5,6,], Christian V. Lundgaard [1], Salome Veshaguri [1], Asger Tønnesen [1], Nikos Hatzakis [1,4], Søren G. F. Rasmussen [2], and Dimitrios Stamou[1,3]**

[1]Department of Chemistry, University of Copenhagen, Universitetsparken 5, DK-2100, Copenhagen, Denmark.

[2]Department of Neuroscience and Pharmacology, Panum, University of Copenhagen, Blegdamsvej 3, DK-2200, Copenhagen, Denmark.

[3]Center for Geometrically Engineered Cellular Systems, Universitetsparken 5, DK-2100, Copenhagen, Denmark.

[4]Department of Chemistry and Nanoscience Center, University of Copenhagen, Universitetsparken 5, DK-2100, Copenhagen, Denmark.

[5]Institute of Biotechnology, Life Sciences Center, Vilnius University, Saulėtekio Ave. 7, LT-10257, Vilnius, Lithuania.

[6]Department of Molecular Compound Physics, Center for Physical Sciences and Technology, Saulėtekio Ave. 3, LT-10257, Vilnius, Lithuania.

*e-mail: marijonas.tutkus@gmc.vu.lt , stamou@chem.ku.dk

MATERIALS AND METHODS

Absorption, Steady-State, Time-Resolved Fluorescence. Absorption spectra were measured by using a Jasco V-670 spectrophotometer. Fluorescence spectra and time-resolved fluorescence were recorded with a Edinburgh-F900 (Edinburgh Instruments) fluorimeter. Excitation of BODIPY493/503 was performed by using a picosecond pulsed diode laser EPL-470 (Edinburgh Instruments), emitting at 473 nm at 1 MHz frequency. Fluorescence decays were measured using the time-correlated single-photon counting technique. Fluorescence decays had 5000 counts at the peak of the decay, with 50 ns windows being used with 4096 channels. Absorption and fluorescence measurements were performed using quartz cuvettes (10 mm). The concentration of dye was kept at 2 μM.

RESULTS

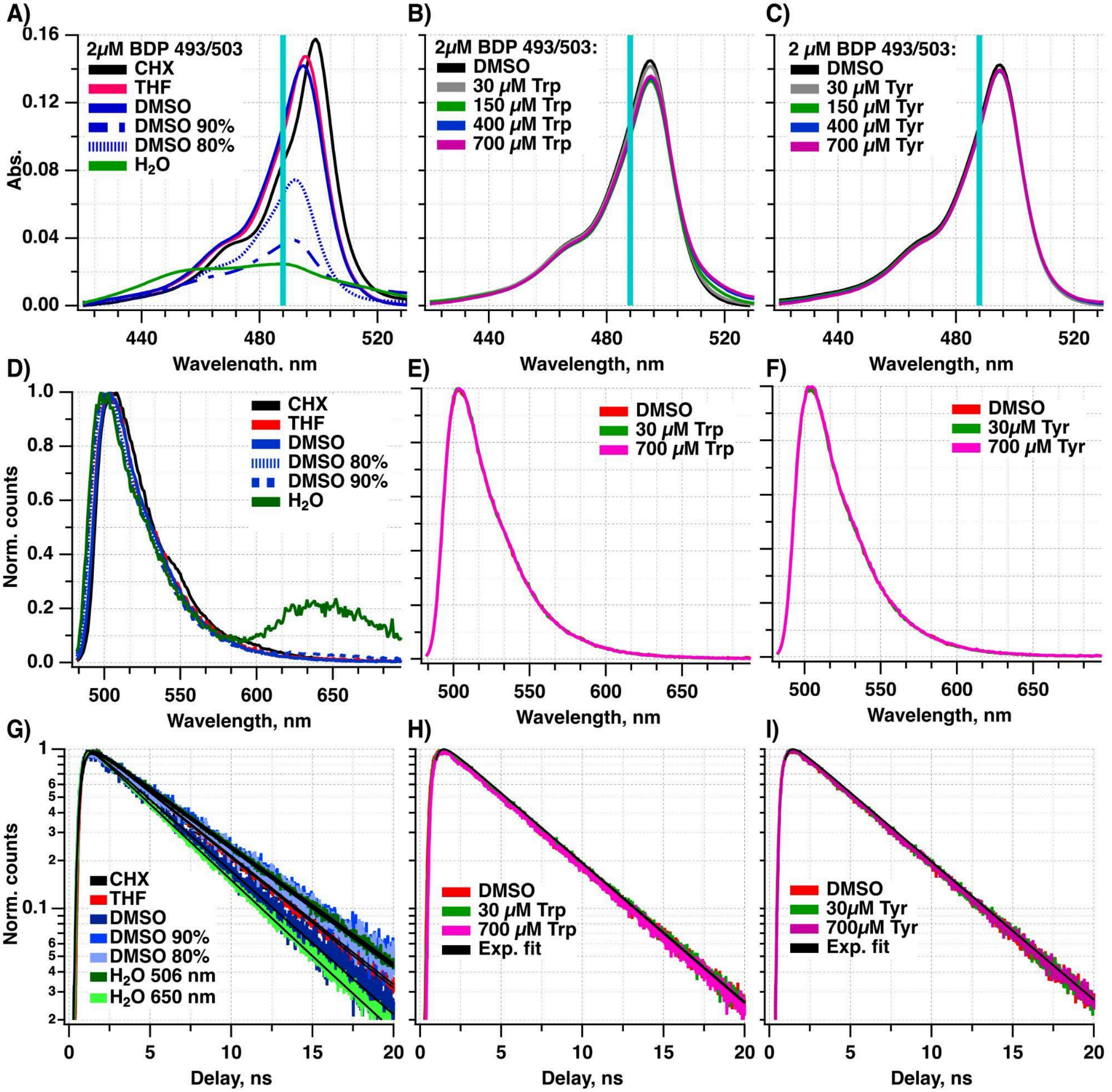

Figure S1. Absorbance (**A-C**) and fluorescence emission (**D-F**) spectra, and time-resolved fluorescence decays (**G-I**) of BODIPY493/503, and their sensitivity to polarity (**A, D, G**) and Tryptophan (Trp) (**B, E, H**) or Tyrosine (Tyr) (**C, F, I**). A fluorescence lifetime (τ) was extracted from time-resolved fluorescence decays by single exponential function fitting (reconvolution using instrument response function). **G)** For Cyclohexane (CHX) τ ~5.4 ns, tetrahydrofuran (THF) τ ~5.3 ns, Dymetilsulfoxide (DMSO) τ ~4.9 ns, 90 % DMSO τ ~5.9 ns, 80 % DMSO τ ~5.9 ns, H_2O (at 506 nm) τ~ 5.9 ns, H_2O (at 650 nm) τ ~4.5 ns. **H)** For 30 μM Trp τ ~5 ns, 700 μM Trp τ ~4.9 ns. **I)** For 30 μM Tyr τ ~5ns, 700 μM Tyr τ ~5 ns. The light blue

vertical line in panels **A-C** indicated the excitation wavelength that we were using in our single-molecule experiments to excite BODIPY dyes.

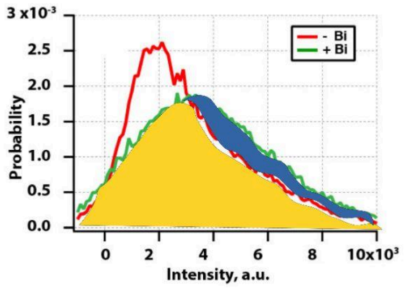

Figure S2. Vertical line-scans of single-molecule (SM) population plots from 0 till 10 seconds for β_2AR-BODIPY 493/503 conjugates (see Fig. 2H). The area marked in blue was divided by the area marked in yellow colour to calculate the average change in intensity from SM signals.
